# Nanopore targeted sequencing in lower respiratory infections: a retrospective study on diagnostic applications, clinical characterization, and antimicrobial guidance

**DOI:** 10.3389/fcimb.2025.1660347

**Published:** 2025-11-07

**Authors:** Qian Chen, Yifan Qiu, Jintao Zhang, Shilong Chen, Xinjun Han, Qingshi Zeng, Guanghai Wang, Xiang Ji, Liang Dong

**Affiliations:** 1 Department of Respiratory, Shandong Qianfoshan Hospital, Cheeloo College of Medicine, Shandong University, Jinan, China; 2 Department of Respiratory, The First Affiliated Hospital of Shandong First Medical University & Shandong Provincial Qianfoshan Hospital, Shandong Institute of Respiratory Diseases, Featured Laboratory of Respiratory Immunology and Regenerative Medicine in Universities of Shandong, Jinan Clinical Research Center for Respiratory Disease, Jinan, China; 3 Department of Radiology, The First Affiliated Hospital of Shandong First Medical University & Shandong Provincial Qianfoshan Hospital, Shandong Lung Cancer Institute, Shandong Institute of Neuroimmunology, Jinan, China; 4 Shandong Provincial Key Medical and Health Laboratory of Translational Medicine in Microvascular Aging, The First Affiliated Hospital of Shandong First Medical University & Shandong Province Qianfoshan Hospital, Jinan, Shandong, China

**Keywords:** nanopore targeted sequencing, conventional microbiological tests, lower respiratory infections, bronchoalveolar lavage fluid, antimicrobial resistance

## Abstract

**Objective:**

This study aims to evaluate the pathogen detection and diagnostic performance of Nanopore targeted sequencing (NTS) versus conventional microbiological tests (CMTs) in patients with suspected lower respiratory infections (LRIs). It also explores the clinical characteristics of patients with discrepant detection results and analyzes the clinical significance of antimicrobial resistance (AMR) gene detection using NTS.

**Methods:**

A retrospective analysis was performed on patients with suspected LRIs admitted to the Department of Respiratory and Critical Care Medicine at Shandong Provincial Qianfoshan Hospital from January 2023 to January 2024. Bronchoalveolar lavage fluid (BALF) and sputum samples were collected from enrolled patients and subjected to both CMTs and NTS.

**Results:**

This retrospective study included 70 suspected LRIs patients (66 BALF, 4 sputum samples), with 56 diagnosed as LRIs, 12 as non-infectious diseases, and 2 undetermined. CMTs detected 14 pathogens, while NTS identified 76. NTS showed higher complete (73.21% vs. 16.07%) and partial (23.21% vs. 35.71%) diagnostic rates than CMTs. Diagnostic metrics favored NTS: sensitivity (96.43% vs. 69.64%), NPV (75.00% vs. 32.00%), Youden index (0.464 vs. 0.363), and AUC (0.732 vs. 0.682), though CMTs had higher specificity (66.67% vs. 50.00%) and PPV (90.70% vs. 90.00%). Patients with concordant positive results (CMTs+NTS+) exhibited more severe clinical features and inflammatory markers than NTS-single positive cases, and had higher exposure to invasive procedures—an confirmed independent risk factor. NTS detected 16 resistance genes in 15 patients, with high ESKAPE pathogen coverage.

**Conclusion:**

NTS overcomes the technical limitations of traditional methods for fastidious pathogens (e.g., intracellular bacteria, mycobacteria) and mixed infections, providing robust technical support for precision anti-infective therapy and complex infection pathogen tracing. Notably, NTS is particularly suitable for early diagnosis in patients with mild symptoms or intact immune function. Compared with CMTs, NTS’s highly efficient and sensitive detection capabilities hold significant practical implications for early infection source isolation, nosocomial outbreak prevention, and optimization of antibacterial drug management strategies.

## 1 Introduction

Lower respiratory infections (LRIs) pose a significant public health challenge (World Health Organization, 2025), imposing a substantial disease burden and high mortality risk (GBD 2019 Diseases and Injuries Collaborators, 2020). The etiological landscape of LRIs is highly diverse, encompassing Gram-positive bacteria, Gram-negative bacteria, atypical pathogens, viruses, and fungi (Shoar and Musher, 2020). Multiple studies (GBD 2021 Lower Respiratory Infections and Antimicrobial Resistance Collaborators, 2024; GBD 2015 LRI Collaborators, 2017; Kang et al., 2023) have demonstrated pronounced health inequalities in LRIs, with over 80% of deaths occurring in resource-limited low- and middle-income countries. Additionally, the escalating issue of antimicrobial resistance (AMR) (The Lancet, 2024) exacerbates clinical management challenges for this disease. Therefore, establishing robust pathogen surveillance networks, enabling rapid pathogen identification, and implementing evidence-based antimicrobial stewardship strategies have become core intervention priorities to optimize LRI diagnosis and mitigate the AMR crisis.

Traditional pathogen identification systems (Hilton et al., 2016) primarily rely on conventional culture techniques, polymerase chain reaction (PCR)-based nucleic acid detection, and antigen/antibody immunological assays (Didelot et al., 2012). As the classical “gold standard” for microbial identification, culture methods exhibit significant limitations, including lengthy detection cycles and suboptimal sensitivity—particularly for microorganisms with specific nutritional requirements or stringent growth conditions (Li et al., 2018). Although immunological detection and PCR-based nucleic acid amplification techniques have overcome dependency on culture, offering rapid and precise detection (Hilton et al., 2016), they require *a priori* knowledge of pathogen classification or preliminary etiological hypotheses derived from clinical characteristics to design targeted detection protocols. This can introduce diagnostic bias or delays in complex scenarios involving unknown pathogens or mixed infections (Forbes et al., 2017). Notably, it has been reported that nearly 60% of patients with fatal LRIs lacked a definitive etiological diagnosis at the time of death (Diao et al., 2021).

Over the past decade, Nanopore targeted sequencing (NTS) (Wang et al., 2021) has undergone remarkable advancements, evolving from pioneering exploratory applications to a key technical approach in genomic sequencing research. It now holds a pivotal role in life sciences (Zhang et al., 2024). Multiple clinical studies (Langelier et al., 2018; Li et al., 2021; Moon et al., 2018; Baldan et al., 2021; Guo et al., 2021; Liu et al., 2022; Hendrix et al., 2021) have demonstrated that NTS exhibits exceptional detection efficiency and sensitivity for respiratory pathogens, enabling precise identification of diverse pathogenic microorganisms (Petersen et al., 2019). Additionally, this technology features robust resistance gene detection capabilities, allowing simultaneous analysis of pathogen antimicrobial resistance profiles. With its real-time data output and ultrafast detection cycle, NTS can significantly reduce the time required to adjust initial empirical antibiotic regimens in severe pneumonia patients (Charalampous et al., 2019). As a pathogen diagnostic technology integrating rapid response and comprehensive detection, it offers substantial clinical advantages over traditional methods (Chen and Xu, 2023).

This study systematically evaluates the diagnostic performance, clinical characteristic correlations, and resistance gene detection rates of novel NTS versus conventional microbiological tests (CMTs) in pathogen identification for LRIs. The analysis uses a single-center retrospective cohort (n=70) to optimize antimicrobial stewardship and guide precision anti-infective therapy.

## 2 Materials and methods

### 2.1 Study population

This study retrospectively analyzed patients admitted to the Department of Respiratory and Critical Care Medicine at Shandong Provincial Qianfoshan Hospital from January 2023 to January 2024. A total of 70 patients with suspected LRIs were enrolled, and all had signed the informed consent form. This study was reviewed by the Ethics Committee of Qianfoshan Hospital, and ethical review was waived due to its retrospective nature.

### 2.2 Inclusion criteria

The inclusion criteria for this study were as follows:

- Patients of any age and gender;
- Lower respiratory infection must meet the following criteria: new or worsening focal or diffuse infiltrative imaging findings on chest X-ray or computed tomography (CT), accompanied by clinical manifestations such as new-onset fever, cough, increased sputum production, dyspnea, or hemoptysis;
- Collection of sufficient bronchoalveolar lavage fluid (BALF) or sputum samples;
- Informed consent signed by the patient or their authorized family members.

### 2.3 Exclusion criteria

- Cases with incomplete data;
- Sample-related issues: insufficient sample volume or samples not meeting the quality detection standards of NTS or CMTs.

### 2.4 Samples and laboratory testing

Bronchoscopy was performed on 66 patients after excluding contraindications, and BALF samples were collected from the lesion sites selected based on chest CT imaging. For the 4 patients who had contraindications for bronchoscopy, sputum samples were collected instead. Subsequently, both BALF and sputum samples were subjected to further testing using CMTs and NTS.

### 2.5 CMTs

For BALF samples, multidimensional microbiological testing was conducted. At the morphological level, Gram staining and acid-fast bacilli smear microscopy were performed to preliminarily observe the morphological characteristics of pathogens. In terms of culture testing, bacterial, mycobacterial, and fungal cultures were conducted to isolate potential pathogens using appropriate media and culture conditions. Throat swab samples underwent real-time fluorescent quantitative PCR to identify SARS-CoV-2, influenza A virus, and influenza B virus. Additionally, immunoassay techniques were employed to detect specific antigens or antibodies of Mycoplasma pneumoniae, Chlamydia pneumoniae, respiratory syncytial virus, adenovirus, cytomegalovirus, and Epstein-Barr virus, thereby providing a comprehensive assessment of the spectrum of respiratory pathogens.

### 2.6 NTS

- Core Principle: Double-stranded DNA is unwound to form single strands, which pass through nanopores embedded with transducer proteins (such as α-hemolysin) under the influence of an applied voltage. As five consecutive bases (5-mer) pass through, they cause specific ion current changes due to spatial hindrance effects. These changes are captured by the transducer proteins, and machine learning algorithms convert the current spectra into base sequences.
- Technical Strategy: The Oxford platform is utilized in combination with metagenomic sequencing (unbiased screening of all microbial nucleic acids in the sample) and targeted sequencing (enrichment of clinically high-risk or difficult-to-lyse pathogens). By aligning with pathogen databases and using intelligent algorithms, this approach enables broad-spectrum pathogen identification and efficient detection of specific pathogens.
  To ensure the reproducibility and standardization of the nanopore sequencing workflow, detailed operational parameters and quality control criteria involved in the experiment have been compiled in a supplementary document (**Supplementary File 1**).

### 2.7 Definitive diagnosis

A rigorous clinical diagnosis was used as the reference standard, with the specific process as follows:

- Preliminary Diagnosis: Two experienced respiratory physicians independently evaluated the complete clinical data of the patients, including symptoms, signs, laboratory tests, imaging results, molecular tests, and pathological evidence (if applicable).
- Consensus Determination: If the diagnostic conclusions of the two physicians were consistent, the diagnosis was confirmed as final. Special attention was given to the detected oral colonizing bacteria and viruses, with a strict assessment of their pathogenicity and clinical significance. In cases of disagreement, the process moved to expert arbitration.
- Expert Arbitration: An arbitration panel consisting of three experts not involved in the initial evaluation conducted case discussions and independent reviews, using a majority vote to determine the final diagnosis.

This diagnostic standard integrates multidimensional data to ensure objective and reliable results, providing an authoritative basis for subsequent performance analysis of the tests.

### 2.8 Statistical analysis

Statistical analyses were performed using SPSS 26.0 software (IBM Corporation, Chicago, IL, USA), with receiver operating characteristic (ROC) curves generated for diagnostic performance evaluation. DeLong’s test was used to compare the area under the ROC curve (AUC). Diagnostic efficacy of CMTs and NTS was evaluated by sensitivity, specificity, among others; their 95% confidence interval (CI) were calculated via the Wilson score method. The Bootstrap method was applied to calculate the 95%CI for Youden’s index. Fisher’s exact tests compared their diagnostic/predictive rates, sensitivity, and specificity. All analyses were conducted in R (version 4.4.2). Graphs were created using Origin 2021. All statistical tests were two-sided, and a P-value < 0.05 was considered statistically significant.

## 3 Results

### 3.1 Baseline characteristics of enrolled patients

This study included 70 patients with suspected LRIs admitted to Shandong Provincial Qianfoshan Hospital between January 2023 and January 2024, of whom 52 were male. Patient ages ranged from 15 to 90 years (mean age: 61.14 years). Baseline demographics and laboratory data are presented in **Table 1**. Following treatment, 61 patients demonstrated clinical improvement or complete recovery, whereas 9 patients ultimately died from the disease.

**Table 1:** Baseline characteristics of patients.

Characteristic	
Age (year, median, IQR)	61.14 (52,73.25)
Male (n, %)	52 (74.3)
Female (n, %)	18 (25.7)
Conscious (n, %)	57 (81.4)
Smoking (n, %)	41 (58.6)
Alcohol use (n, %)	32 (45.7)
Invasive procedures (n, %)	16 (22.9)
Immunosuppressive therapy (n, %)	18 (25.7)
Length of stay (day, median, IQR)	13.48 (7.50,18)
Laboratory data	
WBC (×10^9^/L, median, IQR)	9.40 (5.60,11.89)
NEU (×10^9^/L, median, IQR)	7.42 (3.22,10.18)
CRP (mg/L, median, IQR)	87.22 (15.35,132.85)
PCT (ng/mL, median, IQR)	2.42 (0.77,1.98)
ESR (mm/h, median, IQR)	36.76 (13.50,56.25)
ALB (g/L, median, IQR)	35.02 (29.75,40.05)
Comorbidity	
COPD (n, %)	5 (7.14)
Cancer (n, %)	14 (20)
Diabetes (n, %)	15 (21.43)
CVD (n, %)	23 (32.86)
Prognosis	
Cure (n, %)	61 (87.1)
Death (n, %)	9 (12.9)

IQR, interquartile range; WBC, white blood cell; NEU, neutrophil; CRP, C-reactive protein; PCT, procalcitonin; ESR, erythrocyte sedimentation rate; ALB, albumin; COPD, Chronic Obstructive Pulmonary Disease; CVD, Cardiovascular Disease.

### 3.2 Clinical diagnosis of enrolled patients

**Figure 1:**
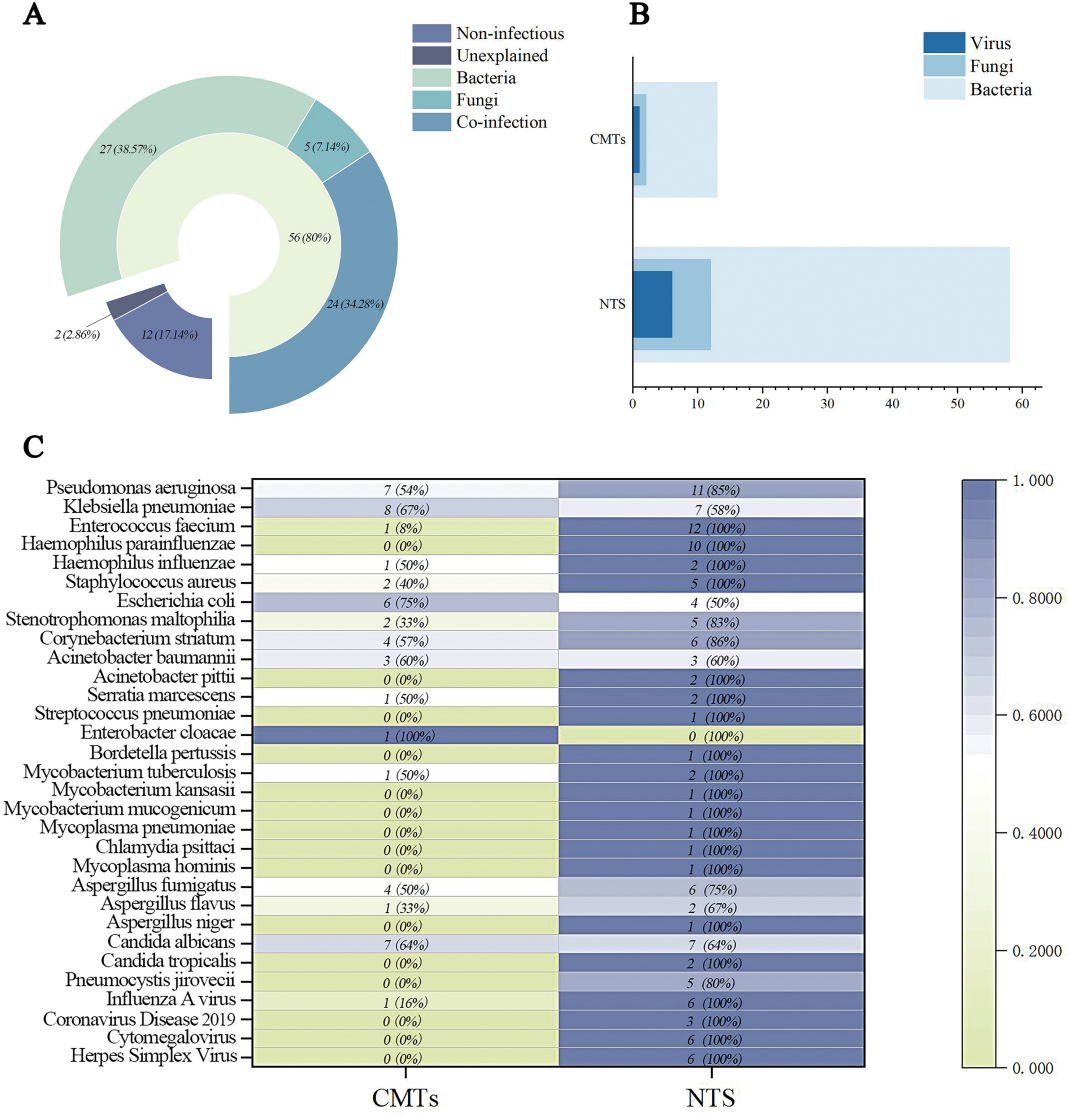
Clinical diagnosis and pathogen detection results of enrolled patients. **(A)** Final Clinical Diagnosis Classification and Distribution of Enrolled Patients. **(B)** The distribution of different classes of pathogens detected in CMTs and NTS. **(C)** Heat map showing pathogen detection profiles of CMTs and NTS. Positive rates calculated against clinically confirmed pathogens (partial bacterial data shown).

Among 70 patients with suspected LRIs, 56 were ultimately diagnosed with LRIs featuring identifiable causative pathogens. Of these, 27 had bacterial pneumonia, 5 fungal pneumonia, and 24 mixed pneumonia. Specifically, 2 cases were confirmed as *Mycobacterium tuberculosis* (MTB) infections, 2 as nontuberculous *Mycobacterium* (NTM) infections, and 3 as atypical pathogen infections. Twelve patients were diagnosed with non-infectious diseases: 2 pulmonary space-occupying lesions, 3 interstitial lung diseases, 2 immune-related pneumonias, 1 lymphoma with pulmonary involvement, 3 organizing pneumonias, and 1 radiation pneumonia. Two cases remained undiagnosed, with unclear etiologies of lower respiratory tract lesions (**Figure 1A**).

### 3.3 Pathogen detection efficacy of CMTs and NTS

CMTs detected 14 infectious pathogens, including 11 bacterial species, 2 fungal species, and 1 viral species. Among these, only *Enterobacter cloacae* was not detected by NTS, whereas the remaining 13 pathogens were successfully identified by NTS. NTS detected 76 infectious pathogens; following clinical validation to exclude non-pathogenic oral commensals and suspected contaminants, 58 bacteria (including 1 MTB, 2 NTM, and 3 atypical pathogens), 12 fungi, and 6 viruses were identified (**Figure 1B**).

In bacterial detection, *Pseudomonas aeruginosa* was the most frequently identified pathogen, with *Enterococcus faecium* and *Klebsiella pneumoniae* ranking second equally, followed by *Haemophilus parainfluenzae* in third place. NTS detected 2 cases of MTB, whereas CMTs identified only 1 case. Notably, NTS identified two NTM species—*Mycobacterium mucogenicum* and *Mycobacterium kansasii*—and three atypical pathogens (*Mycoplasma pneumoniae*, *Mycoplasma hominis*, and *Chlamydia psittaci*) undetected by CMTs. *Candida albicans* was the most prevalent fungus, followed by *Aspergillus fumigatus*—the only two fungal species detected by CMTs. Additionally, NTS’s effectiveness in viral detection provided robust support for antiviral treatment strategies (**Figure 1C**).

### 3.4 Pathogen diagnostic concordance of CMTs and NTS

Among the 56 patients with LRIs, 39 had positive CMTs results. In 9 cases, the pathogens identified by CMTs completely matched the final diagnosis, yielding a complete diagnostic rate of 16.07%. In 20 cases, pathogens were partially detected, with 10 of these cases identifying pathogens considered non-pathogenic, resulting in a partial diagnostic rate of 35.71%. Among the 12 non-infectious patients, 8 had completely negative CMTs results, whereas 4 showed *Staphylococcus epidermidis* and other organisms, which were deemed non-pathogenic following joint evaluation by clinical and radiology physicians.

Among the 56 LRI patients, 54 had positive NTS results. In 41 cases, the pathogens detected by NTS completely matched the final diagnosis, yielding a complete diagnostic rate of 73.21%. Thirteen cases showed partial pathogen detection, with a partial diagnostic rate of 23.21%. One patient with a negative NTS result was ultimately diagnosed with *Pneumocystis jirovecii* pneumonia based on clinical history and imaging, while another was diagnosed with *Pseudomonas aeruginosa* infection based on CMTs results. Among the 12 non-infectious patients, 6 had completely negative NTS results, and 6 showed pathogen detection—predominantly EB virus, herpes simplex virus, and others—which were deemed non-pathogenic following evaluation. The interpretation of viral and oropharyngeal commensal bacteria results from NTS is crucial (**Table 2**).

**Table 2:** Diagnostic concordance rate of CMTs and NTS.

Methods	Partial Diagnostic Concordance Rate	Complete Diagnostic Concordance Rate
CMTs	35.71%	16.07%
NTS	23.21%	73.21%

*Note: 1. Partial diagnostic concordance rate: Refers to the proportion of cases where pathogens detected by CMTs or NTS only cover part of the pathogenic pathogens in the patient’s final clinical etiological diagnosis, and fail to fully match all pathogenic pathogens determined by comprehensive clinical assessment; 2. Complete diagnostic concordance rate: Refers to the proportion of cases where pathogens detected by CMTs or NTS are completely consistent with the patient’s final clinical etiological diagnosis, which is comprehensively determined based on clinical manifestations, imaging examinations, treatment response, and other factors.

**Figure 2:**
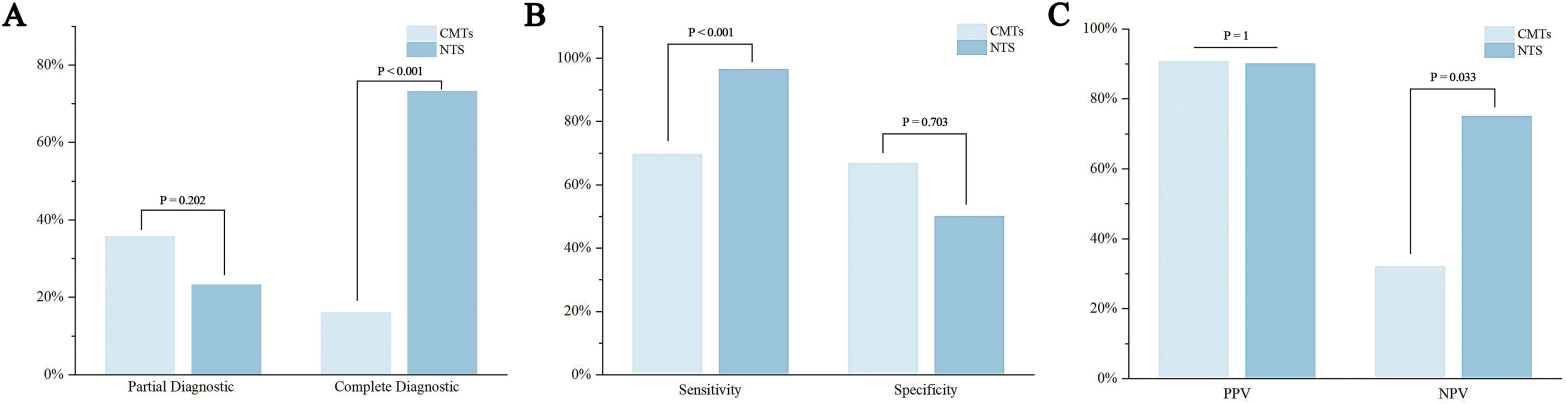
Diagnostic performance and efficacy of CMTs and NTS for pathogen detection. **(A)** Partial and complete diagnostic rates; **(B)** Sensitivity and specificity; **(C)** Positive predictive value (PPV) and negative predictive value (NPV).

Fisher’s exact test revealed a statistically significant difference in the complete diagnostic rate between CMTs and NTS (P < 0.001) (**Figure 2A**).

### 3.5 Pathogen diagnostic efficacy of CMTs and NTS

In this study, the chi-square test results for NTS in infection grouping (infected/not infected) (*χ²* = 20.521, P<0.001) revealed a statistically significant and strong association, demonstrating robust pathogen differentiation capacity and high potential clinical diagnostic value. In contrast, the chi-square value for CMTs (*χ²* = 5.604, P = 0.018) showed statistical significance but a markedly weaker effect size compared to NTS, reflecting the significant limitations of CMTs in diagnostic efficacy. The distribution of diagnostic results is shown in **Table 3**.

**Table 3:** Distribution of diagnostic results for CMTs and NTS in patients.

Methods	Results	Infectious	Non-infectious
CMTs	Positive	39	4
	Negative	17	8
NTS	Positive	54	6
	Negative	2	6

*Notes: 1. “Infectious” represents patients diagnosed as having an infection by the gold standard, and “Non-infectious” represents those diagnosed as not having an infection by the gold standard. 2. “Positive” and “Negative” indicate the test results of CMTs or NTS.

NTS exhibited a sensitivity of 96.43% and specificity of 50.00% for diagnosing LRIs, whereas CMTs showed sensitivities of 69.64% and specificities of 66.67%. The positive predictive value (PPV) and negative predictive value (NPV) of NTS were 90.00% and 75.00%, respectively, compared to CMTs’ PPV of 90.70% and NPV of 32.00% (**Table 4**). Fisher’s exact test revealed statistically significant differences in sensitivity (P<0.001) and NPV (P = 0.033) between CMTs and NTS (**Figures 2B, C**).

**Table 4:** Diagnostic efficacy of CMTs and NTS.

Methods	Sensitivity (95%CI)	Specificity (95%CI)	PPV (95%CI)	NPV (95%CI)	AUC (95%CI)	Youden (95%CI)
CMTs	69.64% (56.18~80.93)	66.67% (38.30~87.67)	90.70% (77.98~97.30)	32.00% (15.95~52.45)	0.682 (0.511~0.852)	0.363 (0.058~0.660)
NTS	96.43% (87.45~99.54)	50.00% (21.07~78.93)	90.00% (79.66~96.23)	75.00% (34.99~96.81)	0.732 (0.546~0.919)	0.464 (0.182~0.750)

PPV, Positive Predictive Value; NPV, Negative Predictive Value; AUC, Area Under the Curve; Youden, Youden Index; CI, Confidence Interval.

**Figure 3:**
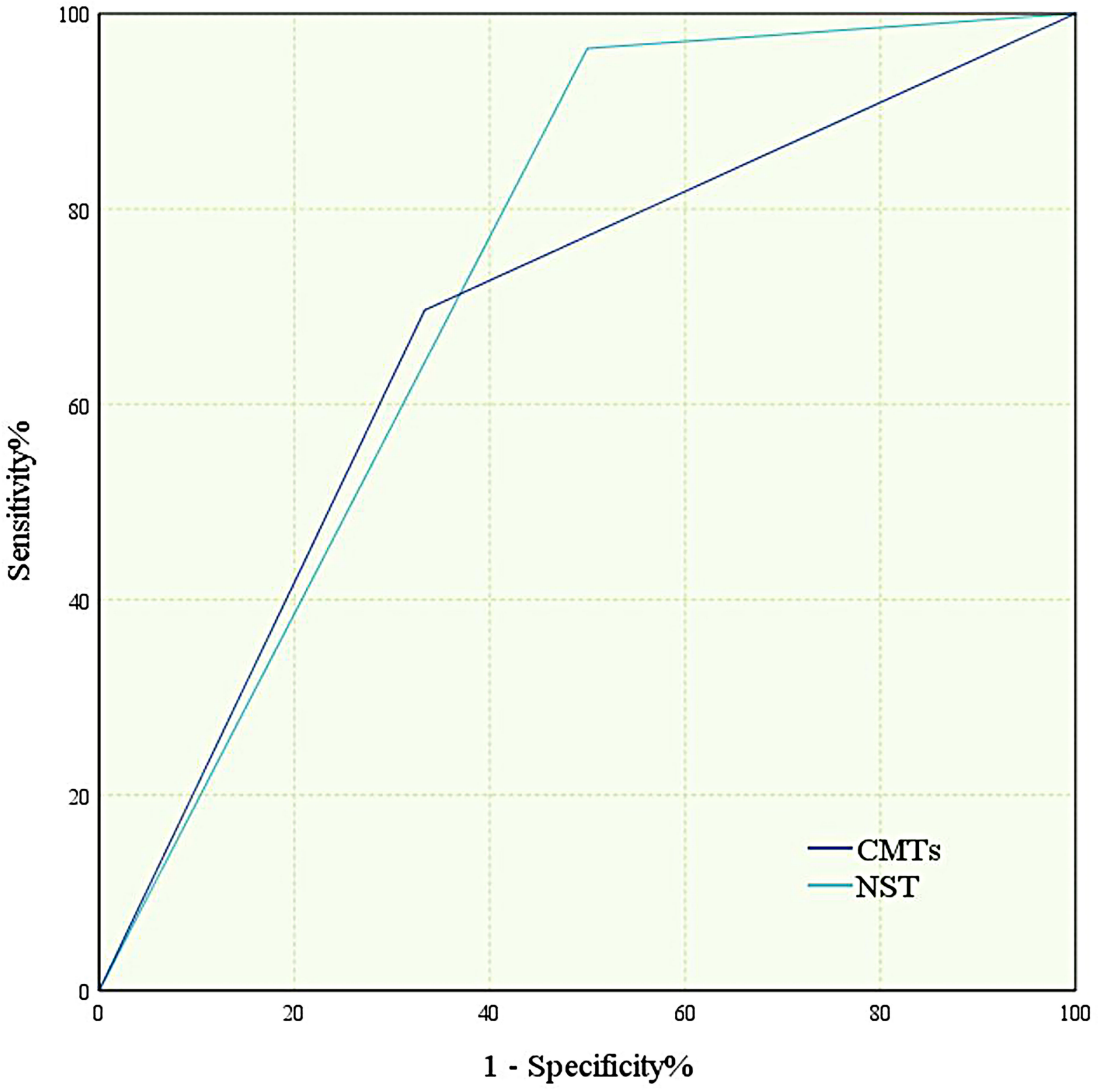
Receiver operating characteristic (ROC) curve of diagnostic efficacy of CMTs and NTS. All ROC curves were constructed based on 56 patients with confirmed infection (gold standard-positive) and 12 patients without infection (gold standard-negative).

The Youden index for CMTs was 0.363, with an AUC of 0.682, while the Youden index for NTS was 0.464, with an AUC of 0.732 (**Table 4**, **Figure 3**). Results of the DeLong test indicated that there was no statistically significant difference in the AUC between NTS and CMTs (*Z* = 0.77974, P = 0.4355).

### 3.6 Differences in clinical characteristics among patient groups

This study included 70 patients, who were grouped based on CMTs and NTS results. The CMTs result was defined as positive (CMTs(+)) if it detected a clinically relevant pathogen; otherwise, it was negative (CMTs(−)). Similarly, NTS positivity (NTS(+)) or negativity (NTS(−)) was determined by pathogen detection. Patients were thus categorized into four groups: CMTs(−)NTS(−), CMTs(+)NTS(−), CMTs(−)NTS(+), and CMTs(+)NTS(+). Specifically, the CMTs(−)NTS(−) group comprised 7 patients, CMTs(+)NTS(−) 2 patients, CMTs(−)NTS(+) 19 patients, and CMTs(+)NTS(+) 42 patients. Considering the sample size and research significance of each group, this study explored clinical characteristic differences between the CMTs(−)NTS(+) and CMTs(+)NTS(+) groups to inform optimal pathogen detection method selection.

**Figure 4:**
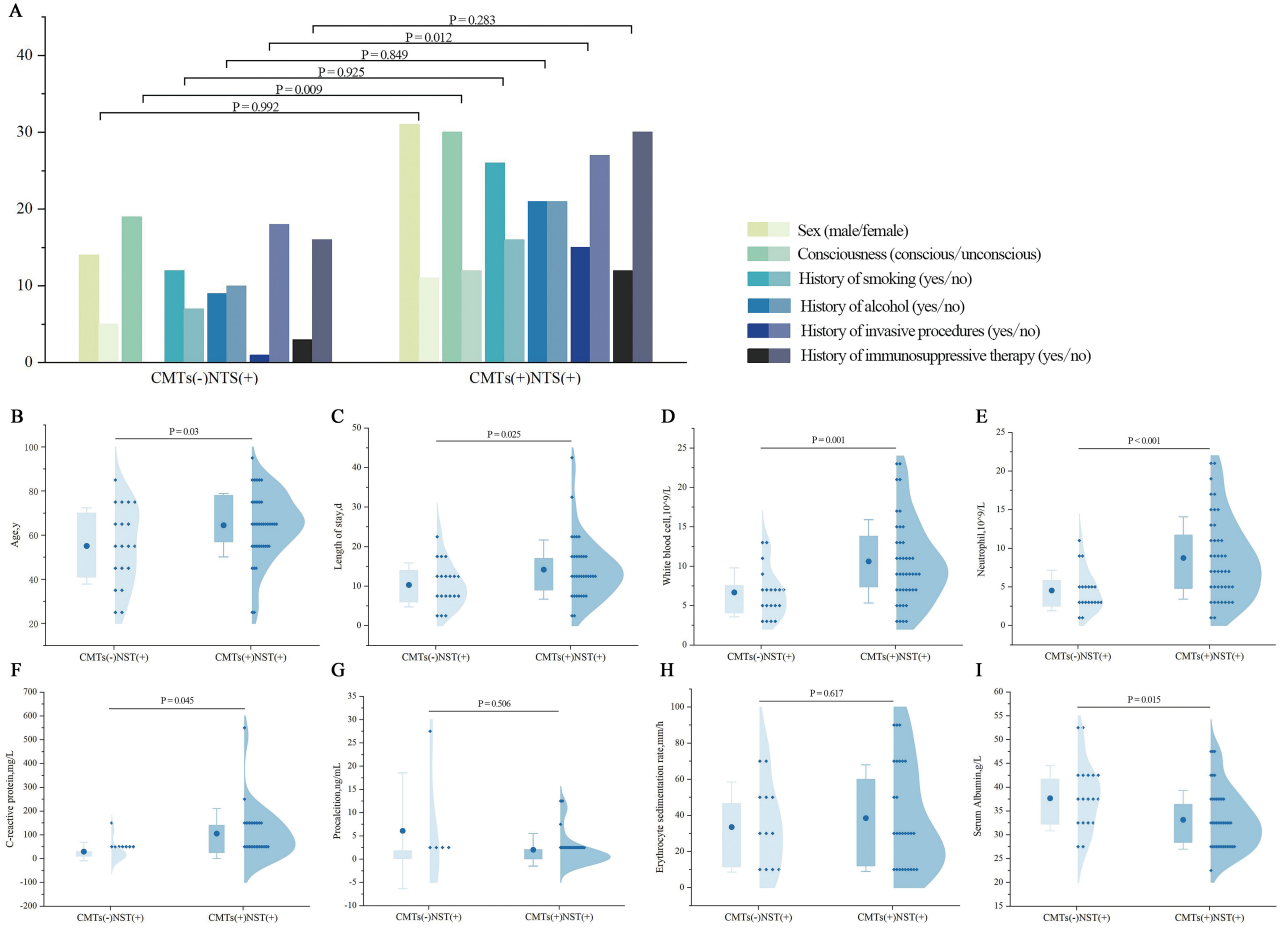
Comparison of clinical characteristics between CMTs(−)NTS(+) and CMTs(+)NTS(+) groups. **(A)** Demographics and medical history: Sex, consciousness, smoking history, alcohol history, invasive procedure history, immunosuppressive therapy history. (X-axis details as labeled; Y-axis: number of patients, n). **(B)** Age; **(C)** Length of hospital stay; **(D)** WBC; **(E)** NEU; **(F)** CRP; **(G)** PCT; **(H)** ESR; **(I)** ALB.

As shown in **Figure 4A**, significant differences were observed in consciousness status and the history of invasive procedures during hospitalization between the CMTs(−)NTS(+) and CMTs(+)NTS(+) groups. The CMTs(+)NTS(+) group exhibited a significantly higher proportion of patients with impaired consciousness than the CMTs(−)NTS(+) group (P = 0.009). Additionally, the proportion of patients who underwent invasive procedures during hospitalization was notably higher in the CMTs(+)NTS(+) group than in the CMTs(−)NTS(+) group (P = 0.012). No significant differences were observed between the two groups in sex, history of smoking, history of alcohol, and history of immunosuppressive therapy.

The CMTs(−)NTS(+) group exhibited significantly younger ages than the CMTs(+)NTS(+) group (P = 0.03). Additionally, the hospital stay duration was notably shorter in the CMTs(−)NTS(+) group (P = 0.025), with statistically significant differences between the two groups. Laboratory data analysis showed no significant differences in procalcitonin (PCT) or erythrocyte sedimentation rate (ESR) levels between groups. However, white blood cell (WBC, P = 0.001), neutrophil (NEU, P < 0.001), and C-reactive protein (CRP, P = 0.045) levels were significantly higher in the CMTs(+)NTS(+) group, whereas albumin (ALB) levels were lower (P = 0.015) (**Figures 4B–I**).

To control for confounding factors including sex, age, smoking history, alcohol history, hospital stay duration, history of immunosuppressive therapy, and consciousness status, multivariate regression analyses were further performed. After adjustment, only WBC (P = 0.032), NEU (P = 0.045), and history of invasive procedures during hospitalization (P = 0.027) remained significantly different between the two groups (**Table 5**); whereas age, hospital stay duration, consciousness status, CRP, and ALB levels—significant in univariate analysis—showed no statistical significance after adjustment (all P > 0.05).

**Table 5:** Multivariate regression results for group differences.

Variable	Effect Size (B/ OR)	95%CI	P
WBC	B=3.197	0.292~6.102	0.032
NEU	B=2.919	0.073~5.764	0.045
Invasive Procedures	OR=0.093	0.011~0.766	0.027

*Note: 1. B: Unstandardized regression coefficient of the independent variable in multiple linear regression. 2. OR: Odds ratio of the independent variable in multiple Logistic regression.

### 3.7 AMR genes and resistance phenotypes identified by NTS

**Figure 5:**
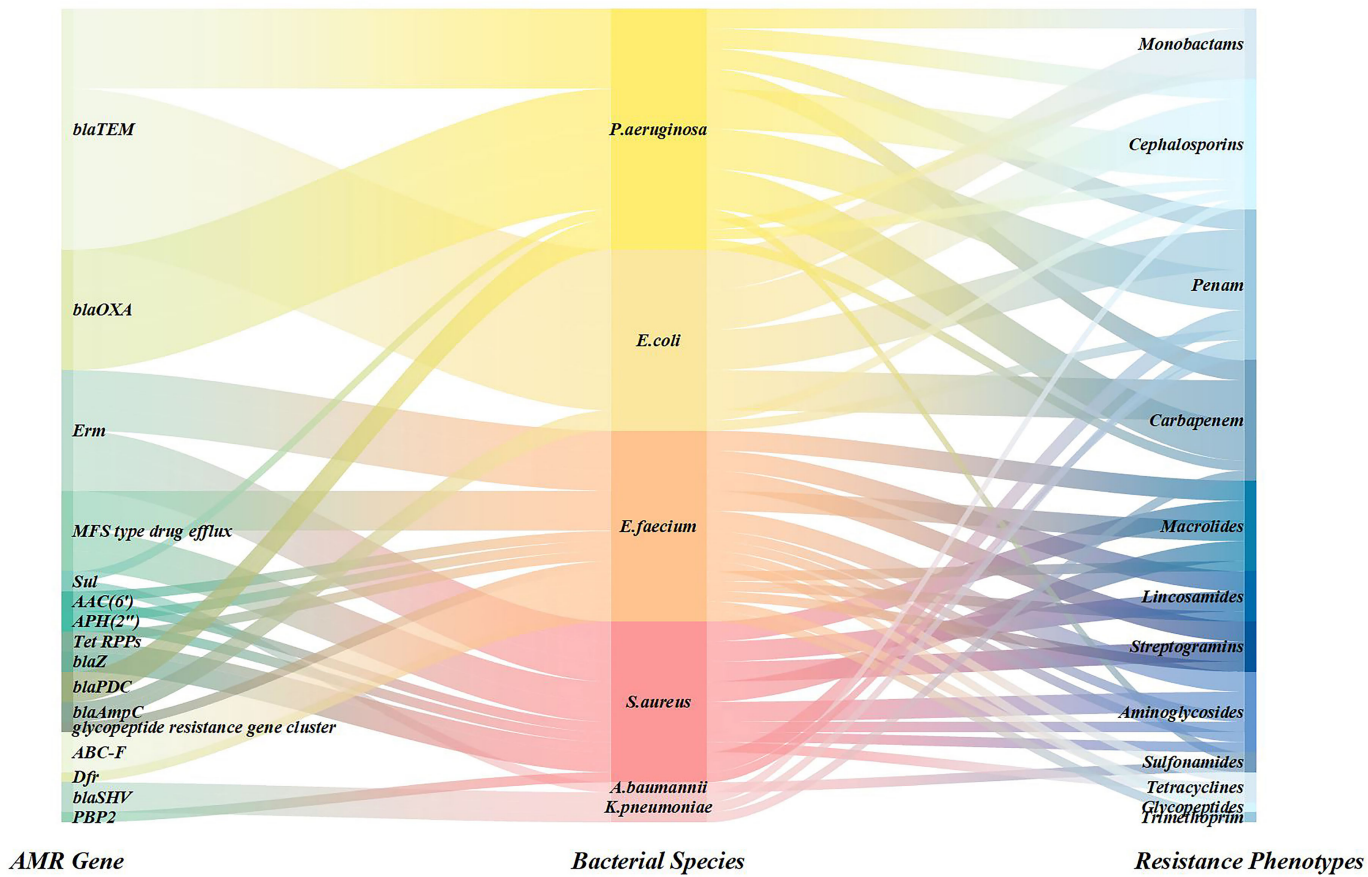
Sankey diagram was used to show the coverage of pathogens identified by NTS and AMR genes.

In this study, 15 of 70 patients had resistance genes detected by NTS, comprising 16 distinct resistance genes. These genes were identified in infections caused by *Pseudomonas aeruginosa*, *Acinetobacter baumannii*, *Escherichia coli*, *Enterococcus faecium*, *Klebsiella pneumoniae*, and *Staphylococcus aureus*. The most frequently detected gene was *bla*TEM (6 detections), followed by *bla*OXA, *Erm*, and MFS-type drug efflux genes (4 detections each). *Sul*, *AAC*(6′), *APH*(2′′), *bla*Z, and *Tet* RPPs were detected twice each, with the remaining genes detected once. Corresponding detection profiles are presented in **Figure 5** (**Supplementary Table S1**).

## 4 Discussion

NTS is a third-generation sequencing technology that combines ultra-multiplex PCR amplification with high-throughput nanopore sequencing (Ciuffreda et al., 2021). It has demonstrated promising clinical utility in the diagnosis of LRIs (Luo et al., 2023). Numerous clinical studies have confirmed that NTS outperforms CMTs in rapid pathogen identification and early diagnostic capability (Zhang et al.,2022a; Ye et al., 2024; Guo et al., 2023; Lin et al., 2024; Fu et al., 2022), which aligns with the conclusions of this study.

NTS detects all major pathogens identified by CMTs, including two NTM species (*Mycobacterium mucogenicum* and *Mycobacterium kansasii*), three atypical pathogens (*Mycoplasma pneumoniae*, *Mycoplasma hominis*, and *Chlamydia psittaci*), and various bacteria, fungi, and viruses missed by CMTs. NTS overcomes the technical limitations of traditional methods in detecting fastidious pathogens (e.g., intracellular bacteria, mycobacteria) and mixed infections (Yu et al., 2025), as it enables rapid and accurate detection of all clinically relevant gene mutations (Wilson et al., 2014), thereby enhancing infectious disease management (Chen et al., 2025a) and control (Sun et al., 2023). These capabilities provide a robust technical basis for precision anti-infective therapy and complex pathogen traceability.

Our study data demonstrate that NTS has a significant advantage in the complete diagnostic rate (73.21%), nearly 4.5-fold higher than CMTs (16.07%). Notably, NTS exhibits a 23.21% partial diagnostic rate, reflecting its adaptability to the varying complexity of clinical diagnostic needs. Multicenter studies (Fu et al., 2022; Zhao et al., 2024; Liu et al., 2024; Yan et al., 2024; Hong et al., 2023) have confirmed that NTS maintains consistent advantages across diverse clinical samples (pleural/peritoneal fluid, BALF, cerebrospinal fluid, urine, blood, wound exudate), with comparable clinical efficacy in LRIs diagnosis. NTS showed significantly higher sensitivity (96.43%) and NPV (75.00%) than CMTs, indicating its capability to substantially reduce missed diagnoses, especially in reliably excluding infections. Although NTS had lower specificity (50.00%) than CMTs (66.67%)—likely due to oropharyngeal commensal bacteria or low-pathogenic microorganism interference—its high PPV (90.00%) and robust pathogen differentiation capacity underscore its core clinical utility. By integrating high sensitivity with reliable negative results, NTS serves as an efficient tool for early precise differentiation of infectious diseases, particularly in complex cases where traditional methods are inconclusive. For diagnostic efficacy metrics, NTS exhibited a Youden index of 0.464 and an AUC of 0.732, outperforming CMTs (Youden index: 0.363; AUC: 0.682). Notably, NTS had an AUC > 0.7, which indicates moderate clinical discriminative ability per Mandrekar (2010), and neither method met the standard for high discriminative ability (AUC > 0.8). Furthermore, the DeLong test revealed no statistically significant difference in AUC between the two approaches (P = 0.4355) —— a result that may stem from the relatively limited sample size in this study. This constraint could reduce statistical test power, thereby failing to detect potential differences in AUC. Collectively, these findings emphasize the need to optimize detection thresholds according to clinical context, minimizing false positives/negatives and facilitating accurate stratified pathogen management.

Clinical characteristic comparisons between groups showed that the CMTs(+)NTS(+) group exhibited more critical clinical features than the CMTs(−)NTS(+) group: a higher proportion of patients with impaired consciousness, a greater rate of invasive procedures, significantly elevated inflammatory markers (WBC, NEU, CRP), and reduced ALB—findings indicative of more severe infection and intense systemic inflammatory response. Conversely, the CMTs(−)NTS(+) group comprised younger patients with shorter hospital stays, suggesting a lower infection burden or effective early intervention. To control for the interference of potential confounding factors, a multivariate regression analysis was conducted. After adjustment, the results showed that WBC (P = 0.032), NEU (P = 0.045), and history of invasive procedures (P = 0.027) remained statistically significant between the two groups. This suggests that infections in CMTs(+)NTS(+) may have triggered a more robust systemic inflammatory response. Additionally, it confirms that invasive procedures themselves may act as “pathogen invasion routes” or “infection risk factors,” directly increasing the probability of pathogen detection by CMTs. This finding offers clear targets for clinical infection prevention and control: for patients undergoing invasive procedures—even younger individuals with normal consciousness—preoperative aseptic management and postoperative etiological monitoring should be enhanced to prevent procedure-related infections from progressing to overt, CMTs-detectable infections.

In summary, NTS overcomes traditional detection limitations by identifying potential pathogens (e.g., low-load or fastidious microorganisms) (Zhao et al., 2024; Chen et al., 2025b), making it particularly suitable for early diagnosis in mild or immunocompetent patients. This enables early targeted therapy, avoids broad-spectrum antibiotics misuse, and reduces resistance risks. For the “history of invasive procedures” as an independent high-risk factor, it is recommended that the early combined application of NTS be implemented. This approach not only improves pathogen detection rates but also leverages NTS’s capability for rapid analysis of drug-resistant genes and strain homology (Wang et al., 2024), thereby facilitating the development of isolation and disinfection protocols for this population at high risk of drug-resistant bacteria transmission and interrupting outbreak chains. Additionally, for NTS single-positive patients with mild inflammatory markers, careful differentiation between colonization and infection is essential to avoid overtreatment and reduce medical costs.

AMR has emerged as a global public health crisis (The Lancet, 2024), with infections caused by ESKAPE pathogens (GBD 2019 Antimicrobial Resistance Collaborators, 2022)—*Enterococcus faecium*, *Staphylococcus aureus*, *Klebsiella pneumoniae*, *Acinetobacter baumannii*, *Pseudomonas aeruginosa*, and *Enterobacter* spp.—being particularly severe (Suzuki et al., 2023). These pathogens not only possess evolutionary mechanisms to evade existing antimicrobial therapies but also exhibit strong transmission capabilities in healthcare settings, leading to a significant increase in treatment failure rates for nosocomial infections. This poses a major challenge to modern medical infection control systems (Antimicrobial Resistance Collaborators, 2022). This study highlights the diagnostic value of NTS by efficiently identifying 16 resistance genes in 15 patients, with a focus on ESKAPE pathogens. NTS can rapidly detect key resistance genes such as *bla*TEM (Farooq et al., 2025) and *bla*OXA (Yoon et al., 2017), clarifying the potential failure risks of carbapenem and β-lactam antibiotics. It also reveals synergistic resistance mechanisms, such as multidrug efflux pumps (MFS) Gaona et al., 2024), providing a basis for the precise selection of reserved drugs like tigecycline and polymyxins. The high efficiency and sensitivity of NTS detection enable real-time tracking of the gene transmission dynamics in these high-risk bacteria (Prior et al., 2025), offering significant practical implications for early infection source isolation, nosocomial outbreak prevention, and optimized antimicrobial stewardship strategies (Bloemen et al., 2025).

This study explored the clinical application value of NTS and CMTs in suspected LRIs patients but has certain limitations. Due to the sample size and heterogeneity (including both BALF and sputum sample types), the evaluation of the two detection methods’ performance may have biases, as BALF samples are less accessible in clinical practice than sputum. Future large-sample multicenter prospective studies are needed to further validate the clinical efficacy of NTS and systematically assess how different sample types impact detection results. Studies have shown that NTS demonstrates superior sensitivity and coverage breadth in detecting bacterial, fungal, viral, and mixed infection pathogens compared to traditional methods, particularly for pathogens not identified by CMTs, thus significantly enhancing the precision of anti-infective treatment. Notably, compared with Illumina-based targeted next-generation sequencing (tNGS) and metagenomic next-generation sequencing (mNGS) —— the more widely used platforms in clinical practice —— NTS offers faster turnaround time and simplified workflows (Xia et al., 2023), both critical for urgent LRIs diagnosis (Zhang et al., 2022b). However, NTS is slightly inferior in per-base accuracy and compatibility with large-scale multiplexed panels; the combination of NTS and Illumina platforms can leverage their respective strengths to enhance the comprehensiveness of detection (Santos et al., 2025). However, this technology still faces challenges such as false positive risks, low detection rates for certain pathogens, and high detection costs. Therefore, clinical practice requires integrating traditional culture results with sequencing data, combined with patient clinical manifestations for integrated judgment, to optimize detection technology application and rationalize clinical decision-making.

## Data Availability

The raw data supporting the conclusions of this article will be made available by the authors, without undue reservation.
