# Supplementary material for: Nanopore targeted sequencing in lower respiratory infections: a retrospective study on diagnostic applications, clinical characterization, and antimicrobial guidance
Source: Front Cell Infect Microbiol. 2025 Nov 7;15:1660347. doi: 10.3389/fcimb.2025.1660347 (PMC12634536; doi:10.3389/fcimb.2025.1660347)
Supplement: Supplementary file 1 [file Table1.docx]

Supplementary Material

**Supplementary Table S1. AMR gene detection of NTS**

| AMR Gene | Bacterial Species (n) | Resistance Phenotypes |
| --- | --- | --- |
| blaTEM | P. aeruginosa(2)、E. coli(4) | Monobactams、Cephalosporins、Penam、Carbapenem |
| blaOXA | P. aeruginosa(4) | Carbapenem、Cephalosporins、Penam |
| Erm | E. faecium(2)、S. aureus(2) | Macrolides、Lincosamides、Streptogramins |
| MFS type drug efflux | E. faecium(2)、S. aureus(2) | Macrolides、Aminoglycosides |
| Sul | P. aeruginosa(1)、A. baumannii(1) | Sulfonamides |
| AAC(6′) | E. faecium(1)、S. aureus(1) | Aminoglycosides |
| APH(2′′) | E. faecium(1)、S. aureus(1) | Aminoglycosides |
| Tet RPPs | E. faecium(1)、S. aureus(1) | Tetracyclines |
| blaZ | S. aureus(2) | Penam |
| blaPDC | P. aeruginosa(1) | Monobactams、Carbapenem、Cephalosporins |
| blaAmpC | E. coli(1) | Cephalosporins、Penam |
| glycopeptide resistance gene cluster | E. faecium(1) | Glycopeptides |
| ABC-F | E. faecium(1) | Macrolides、Lincosamides、Streptogramins、Tetracyclines |
| Dfr | E. faecium(1) | Trimethoprim |
| blaSHV | K. pneumoniae (1) | Carbapenem、Cephalosporins、Penam |
| PBP2 | S. aureus(1) | Penam |

P. aeruginosa: Pseudomonas aeruginosa; E. coli: Escherichia coli; E. faecium: Enterococcus faecium; S. aureus: Staphylococcus aureus; A. baumannii: Acinetobacter baumannii; K. pneumonia: Klebsiella pneumonia.
