# Supplementary material for: Nanopore targeted sequencing in lower respiratory infections: a retrospective study on diagnostic applications, clinical characterization, and antimicrobial guidance
Source: Front Cell Infect Microbiol. 2025 Nov 7;15:1660347. doi: 10.3389/fcimb.2025.1660347 (PMC12634536; doi:10.3389/fcimb.2025.1660347)
Supplement: Supplementary file 2 [file Table2.docx]

Supplementary Material

# Supplementary File 1.

The on-site processing of clinical samples included 3 hours for sample preparation, 1 hour for library construction, and up to a maximum of 4 hours for sequencing and analysis. Sequencing was run until >1000 microbial reads were identified in all the samples on the flow cell (except for the negative control) or to a maximum of 4 hours.

**Host depletion, DNA extraction, and library construction:** BALF (1.5 mL) and sputum (400 μL) samples were collected and stored at 4°C before testing. Sample processing: for host DNA depletion in sputum the final saponin concentration was reduced to 0.1% and HL-SAN DNase volume was reduced to 3μL. The BALF depletion procedure was further optimized by increasing input sample volume to 1.5mL, reducing the HL-SAN DNase to 1μL, and two centrifugation steps were removed. Following host DNA depletion, the final pellet was re-suspended in 750 μL of bacterial lysis buffer (4659180001, Roche Diagnostics, Indianapolis, IN, USA), transferred to a bead-beating tube (Lysis Matrix E, MP Biomedicals, Santa Ana, CA, USA), and bead-beaten at 6 m/s for 40 s in FastPrep-24™ 5G Instrument (MP Biomedicals, Santa Ana, CA, USA). Samples were then centrifuged at 14,000 g for 5 min and DNA was extracted from the supernatant using Maxwell® RSC Whole Blood DNA Kit in Maxwell® RSC instrument (Promega Corp, Madison, WI, USA). DNA quantification was performed using the Quant-it high-sensitivity dsDNA assay kit on Qubit 3.0 Fluorometer (Invitrogen, Waltham, MA, USA). DNA libraries for nanopore-based sequencing were constructed using the Rapid Barcoding Kit (SQK-RBK004, Oxford Nanopore Technologies (ONT) Ltd., Oxford, UK) according to the manufacturer's instructions. Equal quantities of tagmented DNA for up to six samples were pooled and the total library (400 - 900 ng) was loaded on a R9.5 flow cell and sequenced on the GridION X5 platform (ONT Ltd., Oxford UK) according to manufacturer's instructions.

**Real-time sequencing and bioinformatic analysis:** The sequencing process was controlled through ONT MinKNOW software (Version 3.3.2). ONT Guppy (Version 3.0.3) was used for the base-calling of raw sequenced data (fast5 files) into reads (fastq files). Sequencing reads were demultiplexed by blastn-short (Versions 2.7.1+), followed by removal of short (read length ≤ 500 nt) and low-quality reads (mean q-score ≤ 8). Subsequently, host reads were removed by aligning reads to the human reference genome (GRCh38) using Minimap2 (Version 2.14-r883). The remaining reads were assigned to taxonomy using Centrifuge software (Version 1.0.4) and validated by Megablast (Version 2.7.1). Reads with alignments to multiple bacterial species were excluded from further analysis. Abundance was calculated as the number of reads of a microbe divided by the number of total reads of all microbes.

**Definition of Meta-ID:** For species in the common pathogen, the top two most abundant species or all species with abundance over 10% are considered as the Meta-ID; for species not on the list of common pathogens, the top three most abundant species or all species with abundance over 10% were considered as Meta-ID and such thresholds had been proved robust. The following thresholds were applied to remove contamination, misclassification and barcode crosstalk:

1) To reduce barcode crosstalk (misclassification of barcodes during demultiplexing), we excluded Meta-IDs with read number ≤10% of the of the same species from other samples in the same run (e.g. if there were 100 *S. pneumoniae* reads in barcode 2 and 9 *S. pneumoniae* reads in barcode 4, barcode 4 would not be considered positive for *S. pneumoniae*).

2) ONT sequencing produces lower single-read accuracy compared to NGS at an error rate of ~10%, which can lead to the misclassification of related species within the same genus. To reduce such error, we used only reads with unique-alignment and excluded any Meta-IDs with read number ≤10% the read number of a species within the same genus.

3) DNA extraction and library preparation reagents, plastics and the laboratory environment can contaminate the sequencing library with a low abundance species. To overcome this contamination issue, any species with abundance <1% or read number lower than 5 were not considered as Meta-IDs.
